# Why animals construct helical burrows: Construction vs. post‐construction benefits

**DOI:** 10.1002/ece3.11181

**Published:** 2024-09-11

**Authors:** J. Sean Doody, Shivam Shukla, Stephen T. Hasiotis

**Affiliations:** ^1^ Department of Integrative Biology University of South Florida St. Petersburg Florida USA; ^2^ Department of Geology University of Kansas Lawrence Kansas USA

**Keywords:** behavior, cost‐benefit, extended phenotype, helical burrow, helix, ichnotaxa

## Abstract

The extended phenotype of helical burrowing behavior in animals has evolved independently many times since the Cambrian explosion (~540 million years ago [MYA]). A number of hypotheses have been proposed to explain the evolution of helical burrowing in certain taxa, but no study has searched for a general explanation encompassing all taxa. We reviewed helical burrowing in both extant and extinct animals and from the trace fossil record and compiled 10 hypotheses for why animals construct helical burrows, including our own ideas. Of these, six are post‐construction hypotheses—benefits to the creator or offspring, realized after burrow construction—and four are construction hypotheses reflecting direct benefits to the creator during construction. We examine the fit of these hypotheses to a total of 21 extant taxa and ichnotaxa representing 59–184 possible species. Only two hypotheses, antipredator and biomechanical advantage, cannot be rejected for any species (possible in 100% of taxa), but six of the hypotheses cannot be rejected for most species (possible in 86%–100% of taxa): microclimate buffer, reduced falling sediment (soil), anticrowding, and vertical patch. Four of these six are construction hypotheses, raising the possibility that helical burrowing may have evolved without providing post‐construction benefits. Our analysis shows that increased drainage, deposit feeding, microbial farming, and offspring escape cannot explain helical burrowing behavior in the majority of taxa (5%–48%). Overall, the evidence does not support a general explanation for the evolution of helical burrowing in animals. The function and evolution of the helix as an extended phenotype seems to provide different advantages for different taxa in different environments under different physicochemical controls (some traces/tracemakers are discussed in more detail due to their association with body fossils and well‐constrained physicochemical parameters). Although direct tests of many of the hypotheses would be difficult, we nevertheless offer ways to test some of the hypotheses for selected taxa.

## Graphical abstract

Our review revealed helical burrowing in 21 taxa representing 59–184 spp. Helical burrowing behavior has evolved independently many times and only two hypotheses could explain helical burrowing in all species examined. A diversity of explanations may exist for the behavior across taxa.

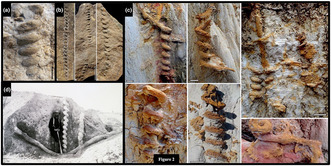

## 1 INTRODUCTION

An extended phenotype, when referring to a single species, includes some architecture or entity (e.g., beaver dam, termite mound) in which the phenotype is the fitness of the construction for survival and reproduction (Dawkins, 1982, 2004). Scientific interest in extended phenotypes has been widespread and sustained, encompassing diverse areas ranging from parasite manipulation of hosts (Hughes & Libersat, 2019) to relationships between genomes and phenotypes (Hunter, 2018) to human sexual selection (Luoto, 2019).

Burrow architectures are extended phenotypes that show great diversity and complexity and can reflect important fitness‐related traits (Hansell & Hansell, 2005; Hasiotis, 2003). In a classic example, the Old‐Field Mouse, *Peromyscus polionotus*, constructs complex burrows with a long entrance tunnel that leads into a nest cavity and a secondary escape tunnel, while its sister species, the Deer Mouse (*P. maniculatus*) builds shorter, single‐tunnel burrows (Weber et al., 2013). The complex burrowing behavior of *P. polionotus* is derived, has a strong genetic component, and its putative adaptive function is to facilitate escape from predation in an open, exposed habitat (Weber et al., 2013; Weber & Hoekstra, 2009; Wolfe & Escher, 1977).

**FIGURE 1:**
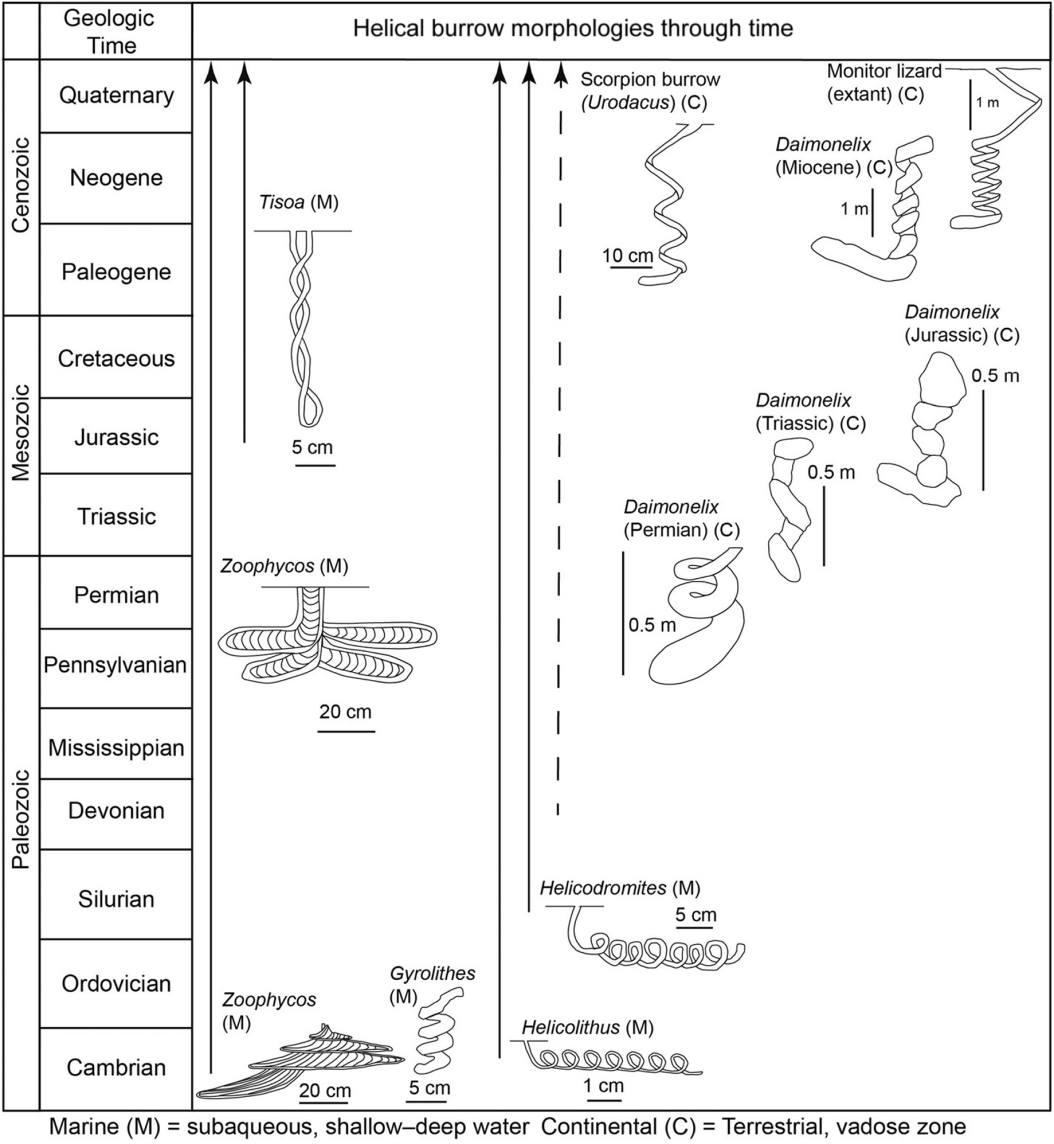
Helical burrow morphologies through the Phanerozoic. Illustrations and ranges based on Häntzschel (1975), Martin and Bennett (1977), Narbonne (1984), Rolfe (1985), Smith (1987), Hasiotis and Bourke (2006), Gingras, Dashtgard, et al. (2008a); Gingras, Pemberton, et al. (2008b), Hasiotis et al. (2013), Uchman and Hanken (2013), Poschmann (2015), Zhang et al. (2015), Fischer and Hasiotis (2018), Raisanen and Hasiotis (2018), Doody et al. (2021), and Wetzel and Blouet (2023).

**FIGURE 2:**
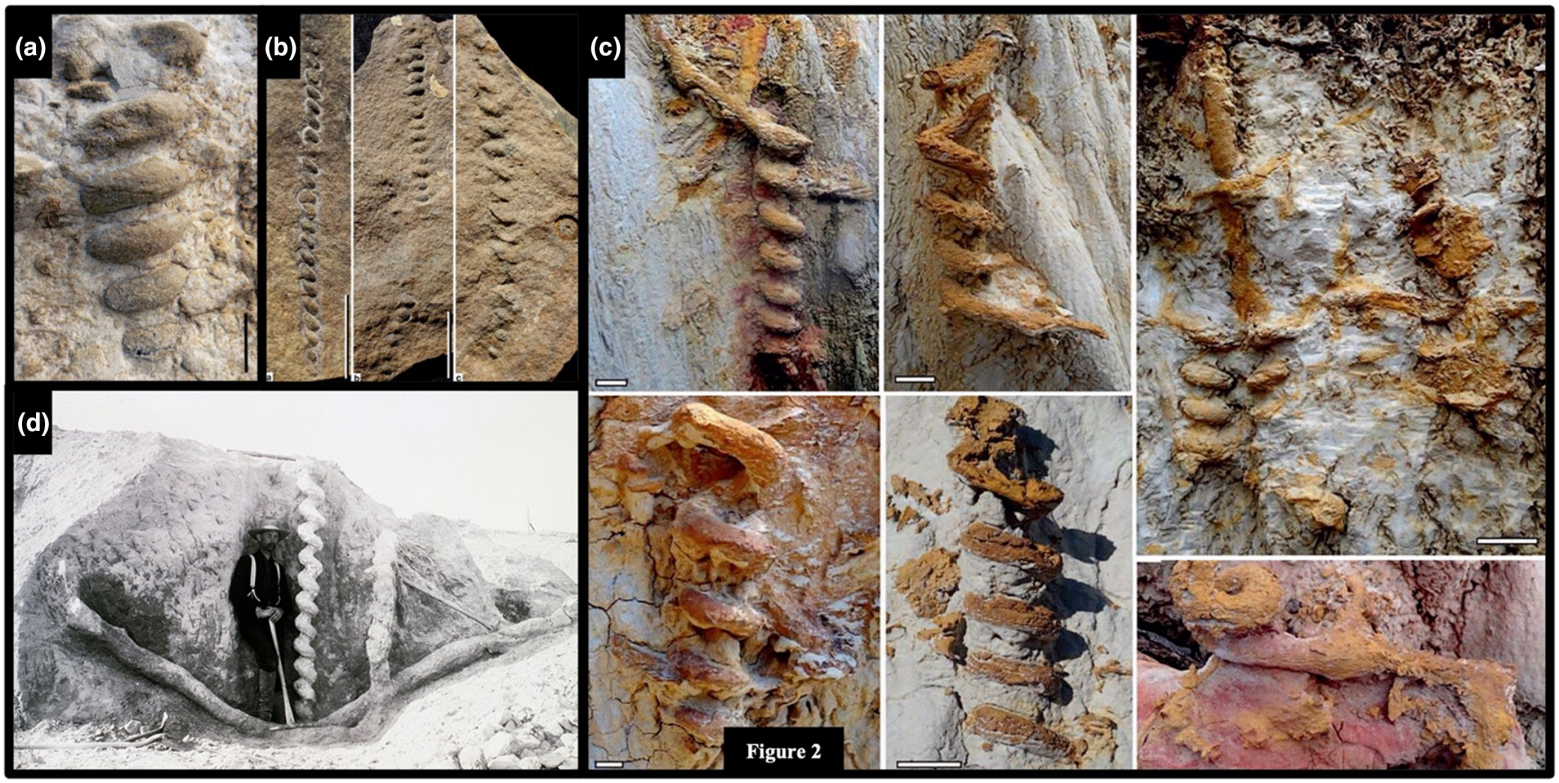
Helical burrows in extinct taxa. (a) *Gyrolithes* probably created by Crustaceans during the Miocene from the Cacela Formation, Portugal (Cachão et al., 2009). (b) *Helicodromites* probably created by vermiform animals during the Devonian from the Hohenrein and Laubach Formations, Germany (Poschmann, 2015). (c) *Gyrolithes* created by shrimp during the Pliocene from the Guadlquivir Basin, Spain (Muñiz & Belaústegui, 2019). (d) *Daimonelix* burrows created by *Paleocaster* during the late Oligocene‐early Miocene in the Harrison Formation, United States (Martin & Bennett, 1977; Permission granted to use, see Doody et al., 2015).

A wide diversity of continental (terrestrial and aquatic) and marine animals excavate enigmatic helical burrows that consist of multiple, asymmetrical to symmetrical whorls descending vertically or laterally to horizontally into a medium (i.e., substrate, sediment, soil). The first of these kinds of burrows, *Gyrolithes* and *Zoophycos*, appeared with the Cambrian explosion ~540 million years ago (MYA), followed later by *Helicolithus* in the late Cambrian and *Helicodromites* in the Silurian (Figures 1 and 2) (e.g., Goldring & Jensen, 1996; Häntzschel, 1975; Hasiotis, 2012; Narbonne, 1984; Poschmann, 2015; Sappenfield et al., 2012; Uchman & Hanken, 2013; Zhang et al., 2015). A study of the macroevolution of *Zoophycos* by Zhang et al. (2015) demonstrated a distinct trend in the size, shape, spreiten pattern, and overall complexity from the Cambrian–Devonian, Carboniferous–Permian, Triassic–Jurassic, Cretaceous, and Paleogene–Quaternary. They were also able to show changes in environmental position from littoral to bathyal settings and tiering depth from shallow to deep. However, in their analysis, Zhang et al. (2015) stated that there is no consensus on the constructor of (the worm‐like sipunculida, echiurida, and polychaeta) or behavior represented by *Zoophycos*, which includes surface‐detritus feeding, refuse dump, cache, deposit‐feeding, and gardening.

**FIGURE 3:**
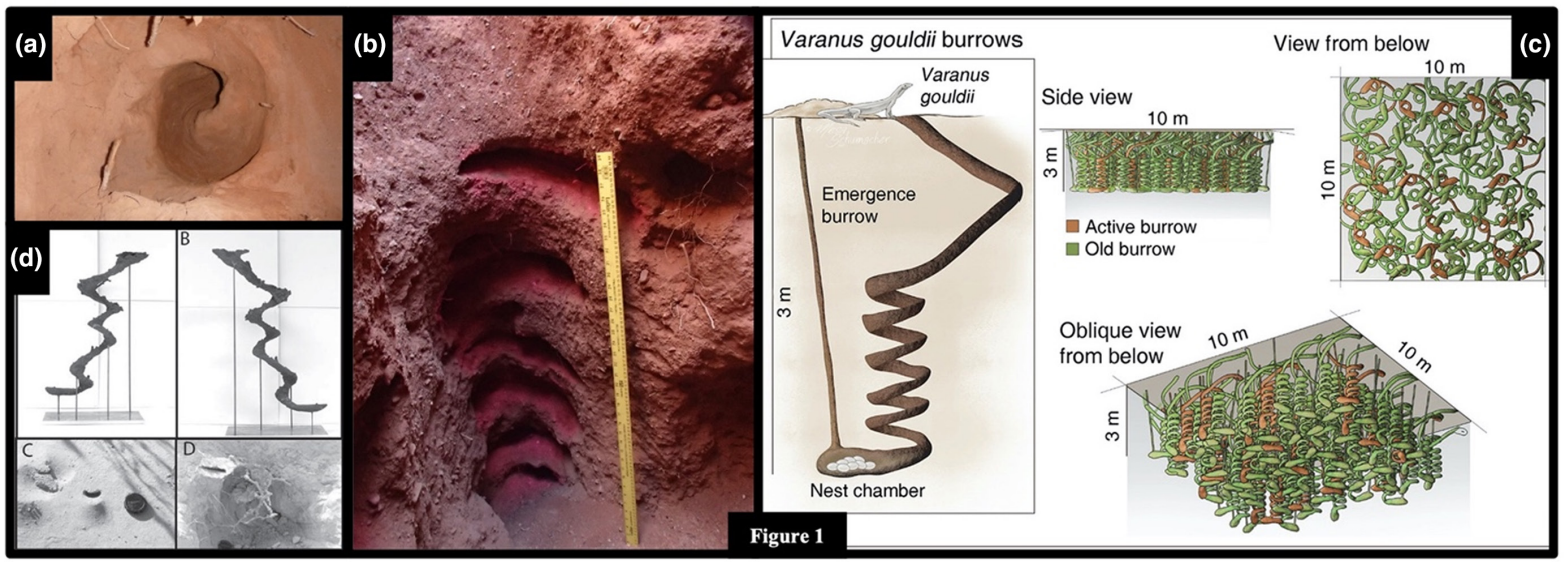
Helical burrows in extant taxa. (a) Top–down view of a *Varanus panoptes* nesting burrow from Australia (J. S. Doody). (b) Side view of a *Varanus gouldii* nesting burrow from Australia (J. S. Doody). (c) Diagram depicting *Varanus gouldii* nesting burrows (Doody, McHenry, Brown, et al., 2018a; Doody, McHenry, Durkin, et al., 2018b). (d) Casts of scorpion burrows (top panels) and entrances (bottom panels) from Australia (Hasiotis & Bourke, 2006).

Many helical burrows are also known only from trace fossils, including the remarkable up to 3‐m‐deep, vertically oriented burrows *Daimonelix* that were constructed by the terrestrial beaver *Palaeocastor* during the Miocene (Figures 1 and 2; Barbour, 1892; Martin & Bennett, 1977). Various forms of *Daimonelix* are now known to have been constructed by a variety of terrestrial vertebrates since the Late Permian ~260 MYA (Fischer & Hasiotis, 2018; Raisanen & Hasiotis, 2018; Smith, 1987). Living examples of species that construct helical burrows include such terrestrial taxa as some pocket gophers, monitor lizards, and scorpions, and such marine forms as some shrimp and some polychaetes (Figure 3; e.g., Doody et al., 2015; Hasiotis & Bourke, 2006; Koch, 1978; Löwemark & Schäfer, 2003; Netto et al., 2007; Powell, 1977; Wilkins & Roberts, 2007).

The marine trace fossils *Helicolithus* and *Helicodromites* (Figures 1 and 2) occur as horizontally oriented, meandering to straight helical burrows, respectively, thought to have been constructed by annelids and/or vermiform animals in marine intertidal to deep water settings (e.g., Häntzschel, 1975; Narbonne, 1984; Poschmann, 2015; Uchman & Rattazzi, 2023). Examples of extant marine species that construct these helical burrows include such taxa as capitellid polychaetes (e.g., *Notomastus*), enteropneusts (e.g., *Saccoglossus*) and nemerteans (Gingras, Dashtgard, et al., 2008a; Gingras, Pemberton, et al., 2008b; Dashtgard & Gingras, 2012).

Other such traces as *Tisoa*—a U‐shaped burrow of a suspension‐feeding vermiform animal originating in the Jurassic (Figure 1)—have recently been described to have a helical burrow pattern produced by the twisting of its U‐shaped tube, sometimes with spreiten between the tubes (e.g., Knaust, 2019; Wetzel & Blouet, 2023). The constructor of this burrow morphology as been suggested to be one or more forms of vermiform animals that used the helical burrow for suspension feeding, deposit feeding, gardening, and/or chemosymbiosis. The occurrence of the helical, U‐shaped spreiten burrow morphology discovered by Wetzel and Blouet (2023) at the type locality of *Tisoa* is significant because the original type material, which was lost, may have included the helical form. Also, this find demonstrates that much is still to be learned from the geologic record in terms of the occurrence, stratigraphic distribution, and helical nature of burrows.

The persistent, independent evolution of helical burrowing behavior across disparate unrelated taxa across hundreds of millions of years attests to its apparent utility and has been the focus of much speculation (e.g., Buatois & Mángano, 2013; Toots, 1963). Toots (1963) hypothesized that the helical nature of such burrows as *Daimonelix* (constructed by vertebrates) and *Gyrolithes* (constructed by enteropneusts, polychaetes, and crustaceans) resulted from the construction by a bilateral symmetrical tracemaker with paired appendages and the influence of gravity acting upon the horizontal excavation to produce an inclined plane and thus a helix. He also pointed out that helical burrows are made in a variety of environments by unrelated groups of organisms. Helical burrows have been proposed to have evolved to: (1) buffer microclimate of the burrow from harsher outside climate conditions (e.g., temperature, seasonality, aridity) in continental settings (Koch, 1978; Martin & Bennett, 1977; Meyer, 1999; Raisanen & Hasiotis, 2018); (2) withstand fluctuating salinity (de Gibert et al., 2012; Laing et al., 2018; Netto et al., 2007; Wetzel et al., 2010); (3) promote drainage during flooding (Doody et al., 2015; Koch, 1978); (4) thwart predators (Doody et al., 2015; Felder, 2001; Gingras, Dashtgard, et al., 2008a; Gingras, Pemberton, et al., 2008b); (5) reduce burrow interference with conspecifics (Gingras, Dashtgard, et al., 2008a; Gingras, Pemberton, et al., 2008b; Doody et al., 2015; Doody, McHenry, Brown, et al., 2018a; Doody, McHenry, Durkin, et al., 2018b); (6) increase the surface area to expose more sediment for deposit feeding (Dworschak & Rodrigues, 1997; Felder, 2001; Pervesler & Hohenegger, 2006); and (7) anchoring (Gingras, Dashtgard, et al., 2008a; Gingras, Pemberton, et al., 2008b); or (8) promote bacterial farming (de Gibert et al., 2012; Dworschak & Rodrigues, 1997; Felder, 2001; Laing et al., 2018; Muñiz & Belaústegui, 2019; Netto et al., 2007). Others, such as Uchman and Hanken (2013), suggested that there is a positive correlation between the morphometrics of *Gyrolithes* and paleoenvironment in which it occurs, with smaller forms reflecting stressed marine environments. Moreover, some have hypothesized that helical burrows could serve multiple functions (Felder, 2001; Neto de Carvalho and Baucon, 2010; Netto et al., 2007; Raisanen & Hasiotis, 2018), fitting into the behavioral category polychresichnia (Hasiotis, 2003).

Yet, the explanation(s) for the evolution and utility of the helix from simple horizontal and vertical burrows remain unresolved. One problem is that little is known of the natural history of the constructors of the fossil burrows (e.g., Bromley, 1996; Hasiotis, 2007; Seilacher, 2007). Another problem is that helical burrows are found in environments representing fully marine to fully terrestrial (continental) settings, produced under vastly different physicochemical conditions (e.g., Bromley, 1996; Gingras, Dashtgard, et al., 2008a; Gingras, Pemberton, et al., 2008b; Hasiotis et al., 2013; Hasiotis & Platt, 2012; MacEachern et al., 2007; Savrda & Bottjer, 1991). Overall, there is no study that has considered all taxa in the search for a general explanation, although a diversity of functions is certainly possible and is sometimes suggested.

Hypotheses offered to explain helical burrowing behavior generally invoke adaptation in the form of “post‐construction” benefits to the creator. Indeed, the adaptive function(s) of helical burrows seems plausible, given their multiple origins in multiple environments, each with its own physicochemical controls, and the increased effort required to create a helix compared to a simpler (straight) burrow of the same incline (volumetric calculations by Meyer, 1999). Much less attention has been given to “construction” costs and benefits, i.e., helical burrows, or those that provide no benefit to the occupant after burrow construction, but rather are restricted to the cost and benefits of burrowing behavior itself. In an exception, White (2001) estimated the total cost of helical burrow construction in scorpions by calculating the net cost of soil transport and the costs of the animal moving itself and soil horizontally, and vertically against gravity. Helically burrowing scorpions minimized both (1) the energy used during burrow excavation by descending as steeply as possible, and (2) the energy required for burrow maintenance, by constructing an entrance run that was shallower than the angle of repose of dune sand (White, 2001). We could not find a similar study conducted on marine animals that construct helical burrows. However, Dorgan et al. (2011), who examined the energetics of burrowing by the cirratulid polychaete *Cirriformia moorei*, which produces crack‐shaped burrows via fracture propagation, found that the energy to burrow, derived from aerobic or anaerobic sources, is not a substantial component of the total metabolic energy, likely incurring a low cost per unit of time. Specific research on helically burrowing annelids, vermiform animals, and crustaceans are necessary to determine if helical burrowing is as beneficial to the burrow producer as is fracture propagation burrowing.

Recently, Doody et al. (2014, 2015, 2021); Doody, McHenry, Brown, et al. (2018a); Doody, McHenry, Durkin, et al. (2018b) found that two species of monitor lizards excavate deep, helical burrows for the sole purpose of nesting and discussed the fit of some post‐construction (adaptive) hypotheses for the function of the helix in these lizards. The communal nests of the yellow‐spotted monitor (*Varanus panoptes*) and Gould's monitor (*V. gouldii*) are by far the deepest extant vertebrate ground nests known, averaging 2–3 m deep and reaching 4 m deep. The soil‐filled burrows consist of an incline to a depth > 1 m, followed by 2–7 tightly descending spirals that terminate in a slightly enlarged nest chamber (Figure 3). Mothers excavate the burrows, lay their eggs, and then abandon the burrows. Unlike scorpions, the lizards do not transport the soil out from the burrows; they remain soil‐filled and the lizards “swim” through the excavated soil after laying eggs. Thus, White's (2001) calculations and conclusions for scorpions and potentially other animals do not apply to the lizards.

Herein we generate and review the major “post‐construction” and “construction” hypotheses for why animals evolve the extended phenotype of helical burrows, with a focus on some continental burrows and burrowers because of their association with body fossils and well‐constrained physicochemical parameters. We address 10 hypotheses including those extracted from the literature and our own. We ask if any of the hypotheses could be general for all taxa. If not, we ask the opposite question: Why did helical burrowing evolve for different reasons in different taxa? To address these two overarching questions, we examine the fit of each hypothesis to each of 21 taxa representing 77–188 species, based on natural history, behavior, and deductive reasoning, from published sources. We outline potential future tests of hypotheses for selected taxa.

## 2 MATERIALS AND METHODS

We surveyed the scientific literature for the evidence of helical burrows in invertebrates and vertebrates using forward and backward searches on Google Scholar and Google (general web search). We excluded species for which there is only one (or less) spiral turn or whorl (e.g., Basan & Frey, 1977; Hembree, 2009, 2014; Kinlaw & Grasmueck, 2012; Linsenmair, 1967; Mikuś & Uchman, 2013; Neto de Carvalho and Baucon, 2010; Paul et al., 2019; Vazirianzadeh et al., 2017). We also excluded studies describing burrows that are weakly helical, weakly sinusoidal or “loosely spiraling” (e.g., Finlayson 1935 in Coelho et al., 2000; Johnson, 1989; Kinlaw & Grasmueck, 2012; Koch, 1978); for example, we excluded ant nests with weakly helical shafts between chambers (e.g., Tschinkel, 2004). Although there is likely a continuum of tortuosity, we found a somewhat dichotomous grouping of burrows that are slightly curved vs. repeatedly or regularly helical. We thus included only species with burrows described as “tortuous” or “possessing regularly descending spiral coils,” or a “helix” (e.g., Koch, 1978; Powell, 1977); we excluded spiral burrow morphologies that occur in a single plane. Although the behavior of constructing weakly helical burrows could be important in understanding the evolution of helical burrowing behavior (indeed, *Urodacus* scorpions do both), taxa exhibiting weakly spiral burrowing are too numerous to consider here. Moreover, the degree of burrow sinuosity has not been quantified for most taxa, making interspecific comparisons difficult. We included papers on both extant and extinct helical burrows produced by animals, although the producers of helical burrows—trace fossils reported as ichnotaxa or described in open nomenclature—in the fossil record are usually unknown. For trace fossils, several tracemakers from different species as well as different phyla can produce a similar trace fossil morphology (ichnotaxon); for example, *Daimonelix* (Fischer & Hasiotis, 2018; Raisanen & Hasiotis, 2018) could be made by reptiles, therapsids, and mammals. Thus, our data rows in Table 1 often reflect more than one species. We also do not consider burrows that occur in one horizontal plane, including sinusoidal traces (e.g., *Sinusichnus*; Belaústegui et al., 2014; Soares et al., 2020) and the spiral feeding traces that occur in soft sediment in one surface plane (e.g., the polychaete *Paraonis fulgens*; Risk & Tunnicliffe, 1978).

**TABLE 1:** Hypotheses for helical or spiral burrows across 21 extant and extinct (emboldened) genera and ichnogenera representing 59–184 spp. (see text for explanation of why there is a range in the number of species).

Genus/Ichnogenus (# spp.)	Post‐construction benefits						Construction benefits			
	Anti‐predator	Micro‐climate buffer	Increased drainage	Deposit feeding	Microbial farming	Offspring escape	Reduced falling soil	Anticrowding^y^	Vertical patch^y^	Biomechan. advantage
*Anuroctonus* (2)	Poss.	Poss.	Poss.	n/a	n/a	n/a	Poss.	Poss.	Poss.	Poss.
*Axianassa* (13)	Poss.	Poss.	n/a	Poss.	n/a	n/a	Poss.	Poss.	Poss.	Poss.
*Axiopsis* (1)	Poss.	Poss.	n/a	Poss.	n/a	n/a	Poss.	Poss.	Poss.	Poss.
*Callianassa* (2–47)	Poss.	Poss.	n/a	Poss.	n/a	n/a	Poss.	Poss.	Poss.	Poss.
*Cratogeomys* (1)	Poss.	Poss.	Poss.	unlikely	n/a	n/a	Poss.	Poss.	Poss.	Poss.
***Cylindrichnus* (2)**	**Poss.**	**Poss.**	**n/a**	**Poss.**	**Poss.**	**n/a**	**Poss.**	**Poss.**	**Poss.**	**Poss.**
*Cynomys* ^*x*^ (1)	Poss.	Poss.	Poss.	unlikely	n/a	n/a	Poss.	Poss.	Poss.	Poss.
***Daimonelix* (2–4)**	**Poss.**	**Poss.**	**Poss.**	**unlikely**	**n/a**	**n/a**	**Poss.**	**Poss.**	**Poss.**	**Poss.**
***Diictodon* (1–7)**	**Poss.**	**Poss.**	**Poss.**	**unlikely**	**n/a**	**n/a**	**Poss.**	**Poss.**	**Poss.**	**Poss.**
*Geomys* (3)	Poss.	Poss.	Poss.	unlikely	n/a	n/a	Poss.	Poss.	Poss.	Poss.
***Gyrolithes* (15)**	**Poss.**	**Poss.**	**n/a**	**Poss.**	**Poss.**	**n/a**	**Poss.**	**Poss.**	**Poss.**	**Poss.**
***Helicodromites* (2)**	**Poss.**	**unlikely**	**n/a**	**Poss.**	**Poss.**	**n/a**	**n/a**	**Unlikely**	**n/a**	**Poss.**
***Lapispira* (1)**	**Poss.**	**unlikely**	**n/a**	**Poss.**	**Poss.**	**n/a**	**Poss.**	**Poss.**	**Poss.**	**Poss.**
*Notomastus* (1)	Poss.	Poss.	n/a	Poss.	n/a	n/a	Poss.	Poss.	Poss.	Poss.
*Ocypode* (1)	Poss.	Poss.	n/a	Poss.	n/a	n/a	Poss.	Poss.	Poss.	Poss.
***Ophiomorpha* (1–3)**	**Poss.**	**Poss.**	**n/a**	**Poss.**	**Poss.**	**n/a**	**Poss.**	**Poss.**	**Poss.**	**Poss.**
*Opisthophthalmus* (1–61)	Poss.	Poss.	Poss.	n/a	n/a	n/a	Poss.	Poss.	Poss.	Poss.
***Paleocaster* (1–4)**	**Poss.**	**Poss.**	**Poss.**	**unlikely**	**n/a**	**n/a**	**Poss.**	**Poss.**	**Poss.**	**Poss.**
*Paruroctonus* (1–8)	Poss.	Poss.	Poss.	n/a	n/a	n/a	Poss.	Poss.	Poss.	Poss.
*Urodacus* (5)	Poss.	Poss.	Poss.	n/a	n/a	n/a	Poss.	Poss.	Poss.	Poss.
*Varanus* (2)	Poss.	Unlikely	Unlikely	n/a	n/a	Poss.	Poss.	Poss.	Poss.	Poss.
SCORE:	100%	86%	48%	48%	24%	5%	95%	95%	95%	100%

*Note*: Designations include “poss.” (possible), “unlikely”, or “n/a” (not applicable, not possible, or highly unlikely). Scores = the proportion (%) possible for each taxon. ^*x*^ = Ichnotaxa & modern taxa; ^y^ = could also result in post‐construction benefits. Data based on deductive reasoning and the following sources: *Anuroctonus* (Williams, 1966); *Axianassa* (Dworschak & Rodrigues, 1997; Felder, 2001; Netto et al., 2007); *Axiopsis* (Dworschak & Ott, 1993); *Callianassa* (Atkinson and Nash, 1990; Dworschak & Pervesler, 1988; James et al., 1990; Nickell & Atkinson, 1995); *Cratogeomys* (Roberts et al., 1997; Villa, 1989; Wilkins & Roberts, 2007); *Cylindrichnus* (de Gibert et al., 2006); *Cynomys* (Ceballos and Wilson, 1985; Schultz, 1942; Wood & Wood, 1933); *Daimonelix* (Fischer & Hasiotis, 2018; Raisanen & Hasiotis, 2018); *Diictodon* (King, 1996; Smith, 1987, 1993; Smith et al., 2021); *Geomys* (Brown & Hickman, 1973; Cameron et al., 1988; Wilkins & Roberts, 2007); *Gyrolithes* (Beynon & Pemberton, 1992; Bromley, 1996; Bromley & Frey, 1974; Buatois et al., 2005; de Gibert et al., 2012; Dworschak & Rodrigues, 1997; Ekdale et al., 1984; Gernant, 1972; Hasiotis et al., 2013; Jackson et al., 2016; Laing et al., 2018; Mayoral & Muñiz, 1995, 1998; Moosavizadeh & Knaust, 2021; Muñiz & Belaústegui, 2019; Netto et al., 2007; Oligmueller & Hasiotis, 2022; Powell, 1977; Wetzel et al., 2010); *Helicodromites* (Poschmann, 2015); *Lapispira* (de Gibert et al., 2012; Lanés et al., 2007); *Notomastus* (Powell, 1977); *Ocypode* (Parenzan, 1931, in: Eshky, 1985; Fellows, 1966, in: Vannini, 1980; Schober & Christy, 1993; Clayton, 2005); *Ophiomorpha* (de Gibert et al., 2012; Loope & Dingus, 1999); *Opisthophthalmus* (Lamoral, 1978); *Paleocaster* (Martin & Bennett, 1977; Meyer, 1999); *Paruroctonus* (Bradley, 1988); *Urodacus* (Adams et al., 2016; Koch, 1977, 1978; Shorthouse & Marples, 1980; White, 2001); *Varanus* (Doody et al., 2014, 2015, 2020; Doody, McHenry, Brown, et al., 2018a; Doody, McHenry, Durkin, et al., 2018b).

We compiled hypotheses offered for the function of the helix, against which we could assign the likelihood that the hypothesis fit a particular taxon or ichnotaxon. For this we used “possible”, “unlikely” or “n/a” (not applicable); “n/a” indicated that there was virtually no chance of a fit, based on deductive reasoning. For example, the hatchling escape hypothesis developed for lizards, which proposes that the helix loosens the soil to facilitate hatchlings escaping the burrow through meters of resistant soil, would not be applicable to aquatic species with open burrows. We subjectively assigned “unlikely” if a good fit was improbable for reasons explained in the text. In contrast, with “n/a”, our reckoning of probability could change with the addition of new information or reasoning. The assignment of “possible” indicated a good fit or potential fit based on the available evidence, context, and our reasoning or that of other authors. In many cases, however, the designation of “possible” reflects the difficulty in testing hypotheses; for example, directly testing the antipredator hypothesis for most extinct species is not possible. Thus, we cannot conclusively claim that a hypothesis is general even if it scores “100% possible” for all taxa. Still, by eliminating some taxa for each hypothesis (by assigning “n/a or ‘unlikely’”) we can potentially conclude that some or all of the hypotheses *could* be general for all taxa.

## 3 RESULTS

Table 1 shows the results of the fit of hypotheses for the function of helical burrows to 21 taxa representing 59–184 species. The wide range in the potential number of species reflects both our lack of knowledge of the burrow types in extant and extinct conspecifics and the uncertainty of the species richness of ichnotaxa.

Of the 10 hypotheses, six are post‐construction hypotheses and four are construction hypotheses. Of the 21 taxa, 12 (57%) are extant, eight (38%) are ichnotaxa and one includes both (5%; Table 1).

Two of the hypotheses, antipredator and biomechanical advantage, were designated as “possible” for all taxa (a score of 100%; Table 1). Other high‐scoring hypotheses were the anticrowding (95%), vertical patch (95%), falling sediment (soil) (95%), and microclimate buffer hypotheses (86%). Two hypotheses, deposit feeding and increased drainage, received moderate scores (both 48%) mainly due to the fit of each to only terrestrial or only aquatic animals (Table 1). The remaining two hypotheses, microbial farming and offspring escape, received low scores (24% and 5%, respectively).

Although the sample sizes for each hypothesis precluded statistical comparison, the mean score for construction hypotheses (91.5 ± 2.99% SE; *N* = 4) is still higher than the mean score for post‐construction hypotheses (49.3 ± 14.06% SE; *N* = 6).

## 4 DISCUSSION

Our review reveals that six of the 10 hypotheses for why animal construct helical burrows cannot be confidently rejected for most of the taxa (86%–100% possible; Table 1). These hypotheses range from “indirectly testable” (falling sediment, vertical patch, biomechanical) to “extremely difficult or impossible to test” (antipredator, anticrowding). Interestingly, four of those six hypotheses are “construction” hypotheses, raising the possibility that helical burrowing could save on energy costs associated with constructing a helix without implicating post‐construction adaptive benefits. Our analysis also eliminated some hypotheses as *general* explanations for the behavior; hypotheses involving increased drainage, deposit feeding, microbial farming, and hatchling escape could not explain helical burrowing behavior in the majority of the animals (5%–48%, Table 1). The function and evolution of the helix as an extended phenotype remain unknown but would seem, in some cases, to provide different advantages for different taxa under different physicochemical conditions. In the following sections we discuss the fit of each hypothesis to selected taxa.

### 4.1 Post‐construction hypotheses

#### 4.1.1 Antipredator hypothesis

**FIGURE 4:**
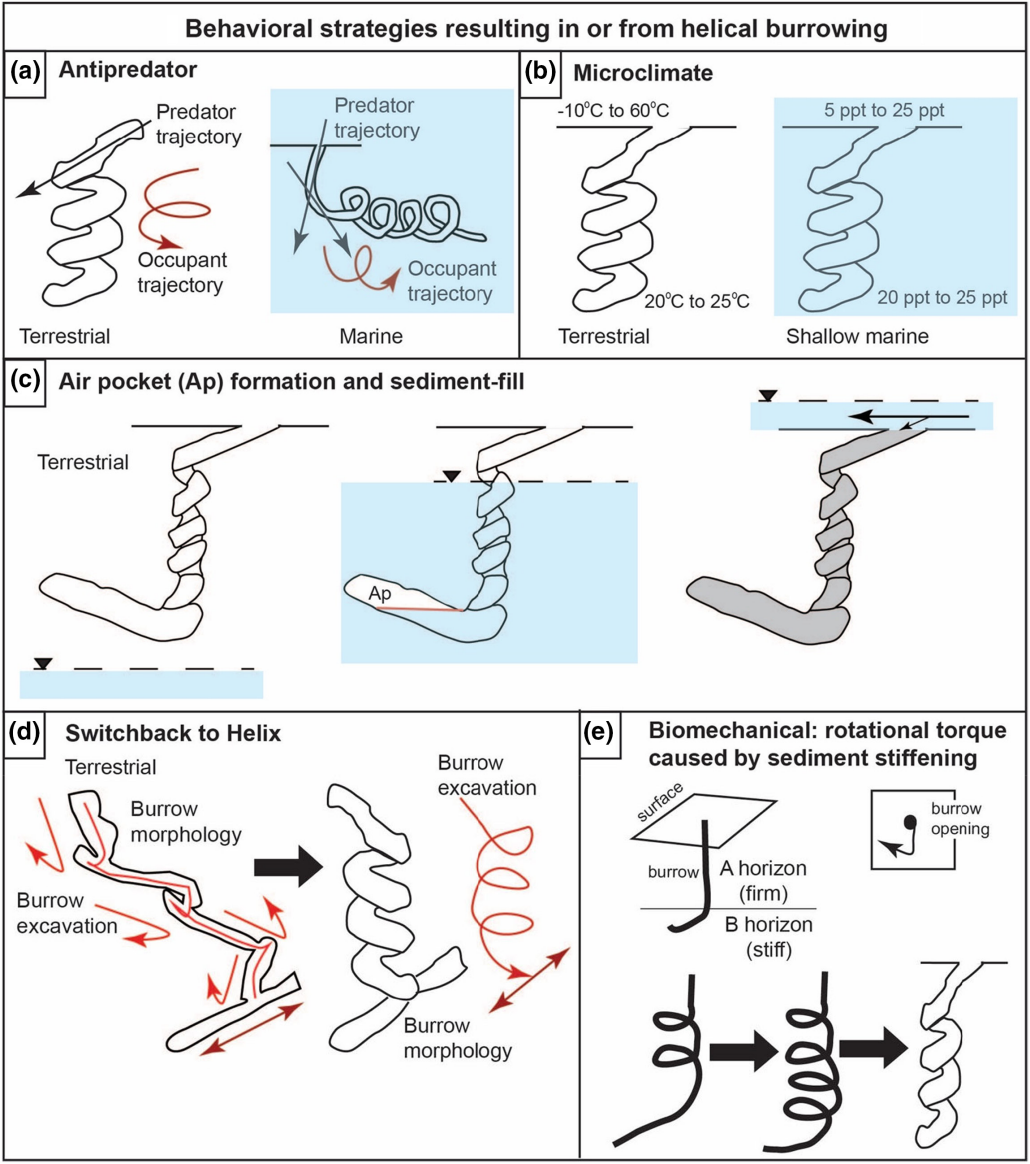
Behavioral strategies resulting in or from helical burrowing. (a) *Antipredator*: the helical burrow pathway misleads or confuses potential predators (black arrow); burrow occupant follows the trajectory of the helical burrow (red arrow). (b) *Microclimate*: the subterranean helical burrow provides near‐constant temperature conditions versus the outside; the marine helix reduces mixing of variable salinity water during ebb and flood tides (blue). (c) *Air pocket formation and sediment‐fill*: the helical burrow during flooding or overland flow fills the burrow and raises the overall water table (blue; dashed line with black triangle), trapping air, producing an air pocket (Ap, white area bounded by red line); sediment‐filled helical burrows trap air in the disturbed sediment pore spaces, preventing flooding of the helix during overland flow (blue; dashed line with black triangle for water table; large black arrow for main flow, small black arrow for very minor flow). (d) *Switchback to helix*: animal excavation in a zig‐zag pattern creates downward, short, J‐shaped shafts (red arrow) to form switchbacks, which intersects with a lateral or inclined tunnel; fully connected J‐shape tunnels produce helices from one level to the other, resulting in a helix or helical burrow with a lateral tunnel at the base, resulting in a helical trajectory. (e) *Biomechanical*—rotational torque caused by sediment stiffening: burrowing downward through a firm horizon (A horizon) into a stiffer horizon (B horizon) causes burrower to turn obliquely or rotate downward while burrowing; continued downward burrowing may produce a helical pattern that through time results in a helical burrow.

Perhaps, the most obvious reason to construct a more complex structure such as a helix, as opposed to a simple structure consisting of single tunnel or shaft, is to reduce the threat of predation of the inhabitant(s) (Doody et al., 2015; Felder, 2001; Martin & Rice, 1994). Indeed, we cannot rule out this explanation for helical burrows of any species in continental or marine environments, (Table 1). Any burrow refuge could decrease predation, but a helix could slow or confuse a predator in pursuit of the inhabitant as it attempts to escape down the burrow (Figure 4a). The helical burrow could also, depending on size and mobility of the predator, prohibit the predator from reaching the inhabitant(s), its eggs, or offspring. For example, the helical burrows of the pocket gopher *Geomys pinetis* may slow down or confuse such predators as weasels or snakes (Brown & Hickman, 1973). Meyer (1999), however, noted that while predators that could fit into *Palaeocastor* burrows (i.e., *Daimonelix*) may have been too long and/or not flexible enough to follow the beaver(s) down into the tight helix, snakes and weasels could easily access helical burrows, and such predators as *Zodiolestes daimonelixensis* (a prehistoric weasel) have been found in fossilized *Palaeocaster* burrows (Martin, 1989; Martin & Rice, 1994). Elsewhere, helical burrows have been speculated to help thwart monitor lizard predators of scorpions (*Urodacus*) (Koch, 1978). Adams et al. (2016) suggested that the cost of predator excavation may increase disproportionately the deeper and more tortuous the burrow, as sand caves in and the tunnel becomes harder to follow.

In the case of monitor lizards (*Varanus panoptes* and *V. gouldii*) that construct helical burrows solely to lay eggs (Figure 3), the helix could thwart egg predators. In support of the predator exclusion geometry, monitor helical burrows are very tight and regular. No egg predators have been identified for these lizards, but the most likely would be conspecific males (Doody et al., 2015; see also Rismiller et al., 2010). Monitor eggs at the burrow terminus would be difficult to detect and reach by any predator because the burrows are 2–4 m deep and soil filled (Doody et al. 2015, 2020; Doody, McHenry, Brown, et al., 2018a; Doody, McHenry, Durkin, et al., 2018b), but, perhaps, the addition of a helix would further frustrate a predatory monitor lizard. Alternatively, Doody et al. (2015) speculated that perhaps the helix in monitor lizards evolved as a deterrent to such now‐extinct predator, as *Thylacinus* or *Megalania* (Clode, 2009).

In marine settings, the helix could serve as an antipredator deterrent below the sediment–water interface as well as with burrows at the sediment–water interface. The helical portions (*Gyrolithes*) of the burrow complex of thalassinidean shrimp *Axianassa australis* have been hypothesized to prohibit the movement of predatory fishes (Dworschak & Rodrigues, 1997; Felder, 2001). Likewise, the small helical burrows of polychaetes (e.g., *Notomastus*) and enteropneusts (e.g., *Saccoglossus*) are strongly associated with anchoring their bodies within their burrows to resist removal by strong currents (Gingras, Dashtgard, et al., 2008a; Gingras, Pemberton, et al., 2008b), but also perhaps as a way to deter predatory fish and crabs from extracting them (Figure 4a).

There is no real evidence for an antipredator function of helical burrows, but testing this hypothesis would be difficult for most species, especially in situ, and not possible for ichnotaxa without modern homologs. In the monitor lizards, field experiments comparing nest predation rates between natural nests and artificial nests without a helix could be a useful indirect test, as could creating baited, artificial helical burrows to see whether (marauding) male monitor lizards could navigate the burrows.

#### 4.1.2 Microclimate buffer hypothesis

An often‐cited potential function of helical burrows is to buffer the burrow microclimate from the outside environment (Figure 4b). Microclimate factors include temperature and humidity in terrestrial environments, particularly those in continental interiors (Adams et al., 2016; Koch, 1978; Martin & Bennett, 1977; Meyer, 1999; Smith, 1987, 1993). Analogous to microclimate buffering in marine settings would be to minimize the effects of fluctuating salinity in tidal zones of shallow marine environments. In our review, this hypothesis cannot be rejected for most taxa (86%, Table 1). Koch (1978) showed that scorpion (*Urodacus*) species in more open areas in more arid parts of Australia construct deeper burrows with more whorls than those that construct burrows under the cover of objects in more mesic areas, concluding “that deep spiral [helical] burrow construction has evolved as an adaptation for the avoidance of harsh surface conditions, and has enabled species of the genus *Urodacus* to spread to otherwise inhospitable arid environments.” While animals constructing deeper burrows in more arid environments to buffer against extreme temperatures and low humidity is logical—Koch (1978) successfully leverages the literature on arthropods in this argument—this apparent relationship does not necessarily have a direct bearing on the presence of, or number of, whorls in a helix, which may simply be a correlate of depth, without implicating microclimate buffering.

The ichnogenus *Daimonelix* has been interpreted as multipurpose burrows (i.e., polychresichnia; Hasiotis, 2003) in which the helix functions to buffer inhabitants from surface extremes in strongly seasonal climates. Noting the seasonally hot and dry paleoclimate inhabited by *Palaeocaster*, the Miocene terrestrial beaver tracemaker of some *Daimonelix*, Martin and Bennett (1977) proposed that the helix would contribute to keeping burrow humidity high. After calculating the comparative volumes and surface areas of helical vs. straight burrows, Meyer (1999) concluded that the *Daimonelix* helical design would have resulted in a more consistent temperature and humidity when extreme variations were experienced at the surface. In support, Smith (1987, 1993; see also Smith et al., 2021) hypothesized that the helical burrows of *Diictodon*, a mammal‐like therapsid from the Permian of South Africa and another *Daimonelix* tracemaker, offered cool, moist burrow climate during extremely hot and dry atmospheric conditions; limited air flow of the helix would allow the humidity of the terminal chamber to rise, especially if near the water table. *Daimonelix* constructed by Late Triassic therapsids and Late Jurassic mammals also occurred in floodplain and alluvial plain settings formed under Late Triassic megamonsoonal and Late Jurassic tropical wet–dry climates (Hasiotis, 2004, 2008; Fischer & Hasiotis, 2018; Raisanen & Hasiotis, 2018). Thus, the hypotheses of microclimate mediation and predator avoidance would apply to these Mesozoic burrows as well.

According to Adams et al. (2016), Koch's (1978) assertion that scorpion burrows are more helical and deeper in drier areas is supported by exposure to more windy conditions and eddies in a turbulent boundary layer on plains and sand dunes (Stull, 1988; Turner & Pinshow, 2015) and moreover by higher rates of water loss in burrowing scorpions than in non‐burrowing species (Gefen & Ar, 2004). Scorpions typically have very low rates of evaporative water loss through their cuticle, however (Hadley, 1970, 1990; Toolson & Hadley, 1977). Thus, helical burrows as an adaptation to sustain high relative humidity, thereby reducing the evaporative water loss of scorpion inhabitants, is plausible.

Interestingly, deep and shallow helical scorpion burrows occur together in central Australia (Hasiotis & Bourke, 2006; Hembree & Hasiotis, 2006). This association indicates that there may be other factors at work, such as: (1) the co‐occurrence of different species with slightly different burrow morphologies; or (2) ontogenetic variation in burrow size, with older and larger scorpions having burrows with larger diameter, depth, and more helical whorls. Also, the orientation of the dune slope with respect to the angle and amount of solar insolation and wind direction on which the burrow is constructed may play a role in the burrow depth, with deeper burrows on north‐facing slopes because of the greater amount of solar insolation.

Some monitor lizards (*Varanus*) construct helical burrows solely for nesting; the 2–4 m deep burrows are unique among helical burrows in that they are soil‐filled (Figure 2; Doody et al., 2014, 2015, 2021; Doody, McHenry, Brown, et al., 2018a; Doody, McHenry, Durkin, et al., 2018b). This places some doubt on microclimate buffering as an explanation for helical burrowing in the lizards (Doody et al., 2015). If climate control is the chief function of the helix, why fill the helix with soil, or why not construct a soil‐filled straight burrow? The answer is not clear. Since the soil‐filled monitor burrows are not inhabited by the lizards themselves, the removal of soil would not be considered when calculating the relative costs of straight vs. helical burrows. Most other nesting monitor lizards construct shallower (<0.5‐m deep) straight burrows in which they remove and then backfill the soil (Pianka & King, 2004); thus, the habit of leaving the soil in the deeper, helical burrows would likely be a derived behavior. Although the deep‐nesting, helically burrowing monitor lizards do remove soil from the first ~0.5 m of the burrow length, the remaining ~1‐3 m of soil is not removed; this action results in the appearance of an apparent abandoned shallow, inclined burrow. Although monitor lizards possibly evolved helical burrow construction in response to dry conditions, and then subsequently adapted to leaving the soil in the burrow to further insulate it or to thwart predators, this sequence of evolutionary events is less parsimonious.

The helical burrows of the ichnogenus *Gyrolithes*, constructed from the Cambrian to the present day, have been interpreted as a refuge from “extreme” salinity fluctuations in transitional environments between the continental and marine realms where tidal variability can be strong (Figure 4b) (Beynon & Pemberton, 1992; Buatois et al., 2005; Netto et al., 2007). Here, marine endobenthic animals experience fluctuating salinities between fully marine to brackish to even fresh‐water conditions depending on fluvial output (Basan & Frey, 1977; Dalrymple & Choi, 2007; Gingras, Dashtgard, et al., 2008a; Gingras, Pemberton, et al., 2008b; Hasiotis et al., 2013). Theoretically, the effect of salinity fluctuations would be diminished because fine sediment of infaunal habitats slows down the exchange of pore water (Rhoads, 1975; Sanders et al., 1965). This hypothesis was based on the idea that *Gyrolithes* was restricted to shallow marine environments interpreted as brackish‐water settings (Gernant, 1972). However, some (e.g., all Cambrian) *Gyrolithes* are found in open‐marine environments, which is suggestive of normal salinity conditions, shedding considerable doubt on salinity buffering as the primary function of the helix (de Gibert et al., 2012; Laing et al., 2018; Netto et al., 2007). Likewise, *Helicolithus* and *Helicodromites* are also known to occur in intertidal to deep marine settings, thus, salinity buffering is not a likely factor (Narbonne, 1984; Poschmann, 2015; Uchman & Rattazzi, 2023). Moosavizadeh and Knaust (2021) questioned the modulation of salinity as the principal function of *Gyrolithes* due to their apparent high‐salinity paleoenvironments. Similarly, *Lapispira*, a double helix burrow, has been found in fully marine deposits (Lanés et al., 2007), shedding doubt on the salinity buffering hypothesis for that ichnotaxon (de Gibert et al., 2012).

There are several important caveats to consider when interpreting behavior and purpose of burrow construction. One of the basic principles in ichnology is that any one particular burrow architecture—in this case, the helical burrows *Gyrolithes*, *Helicolithus*, and *Helicodromites*—can be used for different purposes in different environments under different conditions (e.g., Bromley, 1996; Ekdale et al., 1984). Some of these vertically and horizontally oriented helical burrows occur in the transitional zone where salinities range between marine, brackish, and fresh water, whereas others occur in normal and deep marine settings. Also, important to note is that there are helical burrows assigned to *Gyrolithes* that are components of larger burrow systems, such as *Ophiomorpha* and *Thalassinoides*, which are thought to be used for predator avoidance or several different feeding strategies (see discussions in upcoming sections; Mayoral & Muñiz, 1995, 1998; Dworschak & Rodrigues, 1997; Felder, 2001). The occurrence of *Gyrolithes* has been attributed to brackish‐water conditions but not necessarily extreme in fluctuations, but more like mesohaline or polyhaline salinities. For example, Jackson et al. (2016) and Oligmueller and Hasiotis (2024) described *Gyrolithes* from Lower Permian river‐dominated delta deposits in Antarctica and Upper Cretaceous intertidal deposits in Colorado (USA), respectively. Both of these occurrences are in the transitional zone where salinity fluctuations were a daily phenomenon. Perhaps, the helical design of *Gyrolithes*, *Helicolithus*, and *Helicodromites* would naturally attenuate the exchange of water between the burrow and the overlying tidal water body, much in the same way that *Daimonelix* is thought to limit air flow of the helix to maintain or elevate humidity of the terminal chamber. The amount of water exchanged would be controlled by the animal dwelling within the burrow. Also, the helical structure of *Gyrolithes*, *Helicolithus*, and *Helicodromites* might have been an advantage to the constructor so as not to be hydrodynamically removed from the burrow by changing water currents, or as predator avoidance as the tracemaker withdrew itself into the burrow.

Evidence for the microclimate‐buffering hypothesis is indirect at best for most taxa. In particular, the unique soil‐filled burrows of the monitor lizards raise doubts. Addressing this hypothesis requires understanding which extended phenotype evolved first—the helical or the soil‐filled aspect of the burrow. The two known helically burrowing monitor lizards are sister taxa, and most other species construct simple, inclined, soil‐filled nesting burrows (Pianka and King, 2004). The ancestral burrow morphology for the helical nesting monitor lizards is thus likely to have been soil‐filled (back‐filled or with soil left in place). The helical burrows are also extremely deep (Doody et al., 2014, 2015, 2021; Doody, McHenry, Brown, et al., 2018a; Doody, McHenry, Durkin, et al., 2018b)—another derived trait.

#### 4.1.3 Increased drainage hypothesis

A third hypothesis for helical burrows constructed by taxa in terrestrial environments proposes that the helix provides improved drainage under flood conditions via increased surface area, thereby preventing or reducing burrow flooding that could, for example, cause mortality or expulsion of scorpions or failure of lizard eggs (Doody et al., 2015; Koch, 1978). This explanation is rejected for a majority of taxa (though possible in 48% of taxa, Table 1). Koch (1978) proposed that the extensive spiraling burrows of the scorpion *Urodacus* would reduce the effect of sheet flooding during the wet season. This idea may be supported by seasonal flash flooding apparently experienced by *Diictodon*, the constructor of Permian *Daimonelix* (King, 1996), although this hypothesis was not explicitly discussed (Smith, 1987; Smith et al., 2021). Indeed, somewhat ironically, preservation of the helical burrows in alluvial environments relies on flooding (e.g., Smith et al., 2021).

Although the helix itself may not be beneficial for drainage after flooding, the upturned terminal chambers on many of the Miocene and some of the Jurassic *Daimonelix* (Martin & Bennett, 1977; Raisanen & Hasiotis, 2018) have been thought to trap air in the burrow chamber so that during flooding, the burrower would not drown in its burrow (Figure 4c) (Hasiotis et al., 2004; Raisanen & Hasiotis, 2018).

Nesting in both *V. panoptes* and *V. gouldii* in northern Australia is during the late wet season and early dry season, and there can be substantial rainfall including sheet flooding during the first 2 months of incubation for the earlier nests. Although the lizard burrows are soil‐filled, the soil is somewhat loose early in incubation. The loose soil combined with the increased surface area of the helix could impede water infiltration by trapping air in the soil pores in the fill of the helix (Figure 4c); this would act to improve drainage allowing water to bypass the nest rather than infiltrating it, thereby preventing egg inundation or reducing the amount of time eggs are inundated. Lizard eggs can withstand inundation for up to 6 h based on experimental data (Heger & Fox, 1992; Losos et al., 2003).

#### 4.1.4 Deposit‐feeding hypothesis

Some modern crustaceans (i.e., shrimp), polychaetes, enteropneusts, and other vermiform animals may construct the helical burrow assigned to *Gyrolithes* (e.g., Dworschak & Rodrigues, 1997; Neto de Carvalho and Baucon, 2010; Pervesler & Hohenegger, 2006; Powell, 1977; Gingras, Dashtgard, et al., 2008a; Gingras, Pemberton, et al., 2008b; van der Horst, 1934) for deposit feeding in shallow to deep‐water marine settings. Specifically, the increased surface area of the helix compared to a straight burrow would enhance deposit feeding by optimizing the utilization of nutrients in a given sediment volume in animals. For example, the helices made by the thalassinidean shrimp *Axianassa australis* as part of their burrow complex may allow the animals to burrow to greater depths with gentle slopes in order to exploit deeper sediment layers rich in organic matter (Dworschak & Rodrigues, 1997; see also Atkinson and Nash, 1990; Nickell & Atkinson, 1995 for similar conclusions for the shrimp *Callianassa subterranea*). Although deposit feeding in *A. australis* burrows needs confirmation, the poor fit of the diameter of the shrimp to the burrow diameter suggests deposit feeding, because suspension feeders tend to fit closely into their burrows (Dworschak & Rodrigues, 1997; see also Pervesler & Dworschak, 1985). A close fit is necessary for effective ventilation of the burrow for respiration and feeding in suspension feeders (Dworschak, 1981, 1987). Wetzel et al. (2010) considered deposit feeding as likely in *Gyrolithes*, partly based on the finding of an abundance of plant material in the vicinity of the burrows (Dworschak & Rodrigues, 1997). Laing et al. (2018), however, considered deposit feeding unlikely in *Gyrolithes*, whether made by polychaetes or decapod crustaceans, based on the lack of evidence of active fill or fecal pellets. However, the presence or absence of backfill menisci and/or fecal pellets is not necessary to determine if a burrow is used for deposit feeding. There are many callianassid and other thalassinidean shrimp that produce fecal pellets while in their burrows and expel them from the burrow by recirculating the water (e.g., Curran & Seike, 2016; Kennedy et al., 1969; Netto et al., 2017).

Helical burrows constructed by animals in terrestrial settings are not involved in feeding, based on the lack of food resources deep in the ground, with the exception of roots and tubers, which are shallow and close to the surface, and based on the lack of frequent branching and fecal fillings (Toots, 1963). The common mole rat does construct complex, shallow burrows to feed on roots and tubers, but does not construct helical burrows (e.g., Spinks et al., 2000). Analogous burrow morphologies to these modern burrowers have been found in Lower Jurassic continental erg deposits of the Navajo Sandstone in Utah (Riese et al., 2011).

#### 4.1.5 Microbial farming hypothesis

In a variation of the deposit‐feeding hypothesis, marine burrowers have been hypothesized to create vertically and horizontally oriented helices, such as *Gyrolithes*, *Helicolithus*, and *Helicodromites* (Figures 1, 2), to optimize feeding in organic‐rich zones or anoxic sediment by increasing the sediment‐to‐burrow ratio for the purpose of microbial farming or gardening (e.g., Felder, 2001; Moosavizadeh & Knaust, 2021; Poschmann, 2015; Seilacher, 2007; Wetzel et al., 2010). Netto et al. (2007) argued that Permian and younger *Gyrolithes* were created by crustaceans, rather than by polychaetes, as proposed by Powell (1977) (see also Gingras, Dashtgard, et al., 2008a; Gingras, Pemberton, et al., 2008b); however, ample evidence from studies of modern marine faunas and their comparison to ancient trace fossils demonstrates that *Gyrolithes* can also be constructed by variety vermiform animals as well (e.g., Gingras, Dashtgard, et al., 2008a; Gingras, Pemberton, et al., 2008b; Hauk et al., 2009; Powell, 1977; van der Horst, 1934). A study by Bird et al. (2000) on burrows of the thalassinidean shrimp *Biffarius arenosus* found that the burrow walls contain a greater abundance of microbial consortia compared to the surrounding sediment. This was due to the bioirrigation of the burrow by the shrimp providing oxygenated water into burrow, further extending the sediment–water interface into the subsurface and increasing the redox potential and bacterial growth between the burrow and the sediment. Moosavizadeh and Knaust (2021) considered microbial farming as the most likely function of *Gyrolithes* from Iran, which occurred in soft, low‐energy sediments. Poschmann (2015) hypothesized the function of helical burrows of trace fossil *Helicodromites* as microbial farming under oligotrophic conditions in a well‐oxygenated environment. Extant producers of *Helicodromites* and *Helicolithus* as discussed by Steward et al. (1996) and Kappel (2003) and summarized by Uchman and Rattazzi (2023) appear to be associated with higher numbers of microbes and the anoxic‐oxic chemical gradient between the burrow wall and the matrix may benefit the growth of those microbes. In these situations, the helical burrow morphology may be conducive for microbial farming.

The occurrence of *Tisoa siphonalis* in organic‐rich mudrock, along with its extraordinary depth (>2 m) and its association with pyrite suggests that the producers might have fed on microbes flourishing along the tube wall that also exploited the extreme redox condition (Wetzel & Blouet, 2023). For example, the sulfate reduction zone and methanogenesis zone would have been reached by those burrow depths where oxygenated surface waters were circulated and encourage microbial growth under those conditions. Another possibility is that the tracemaker acquired its nutrition via chemosymbionts living within its gut by up‐taking sulfide and/or methane.

Several issues arise from the relationship between microbial consortium‐burrow wall association and the microbial farming or gardening hypothesis. First, are the animals *actually* utilizing the microbial consortia as a food source? Feeding on microbes growing on burrow walls has been previously proposed, however, it has not been demonstrated yet to your knowledge. Burrow walls have been observed to be smooth and mucus covered because of microbial growth (e.g., Bird et al., 2000; Dworschak & Rodrigues, 1997; Felder, 2001), but there is apparently no direct evidence of the tracemakers feeding on the walls or on the sediment that makes up the walls. Studies of gut contents of selected thalassinidean shrimp via dissection and C and N isotopic analyses (Abed‐Navandi & Dworschak, 2005; Shimoda et al., 2007) showed that a range of live seaweed, seaweed‐derived detritus buried in sediment, live enteromorphs, phytoplankton or fresh phytoplankton‐derived detritus, and benthic microalgae most likely constituted their food source. The smallest organic particles in the ambient sediment around the burrows, together with the burrow‐wall lining, may serve as a nitrogen source for *Neocallichirus grandimana*, but its main carbon source remains unknown. For the species with conclusive diets, the low organic content of tropical littoral sediments may help explain their predominant reliance on more nutritious food sources foraged from the sediment surface. Abed‐Navandi & Dworschak (2005) found that the subsurface areas of the burrows function only as places where food is processed rather than where it is acquired in regard to nutrition. These studies would suggest that thalassinidean shrimp do not use the microbial consortia growing on the burrow walls as a food source, falsifying the microbial farming or gardening hypothesis. Likewise, this may also be the case for vertically and horizontally oriented helical burrows constructed by polychaetes and other vermiform animals.

The growth of the microbial consortia on the burrow walls is a consequence of bioirrigation of the burrow system in order to maintain the oxygenation levels, not for the purpose of gardening or farming. This occurrence has been assumed to be farming or gardening but is merely incidental result.

*Gyrolithes* is often associated with the mazework or boxwork burrow systems assigned to *Thalassinoides*, *Ophiomorpha*, or *Spongeliomorpha* (Dworschak & Rodrigues, 1997; Mayoral & Muñiz, 1995, 1998; Neto de Carvalho and Baucon, 2010), which are interpreted as dwellings used for suspension feeding and deposit feeding (Dworschak & Rodrigues, 1997; Felder, 2001) based on the overall complexity of the system. The combination of architectural elements of the boxwork structure integrated with the helical structures is a compound burrow structure that would be classified as polychresichnia (multipurpose structure) in which dwelling, suspension feeding, deposit feeding, reproduction, and possible “microbial farming” takes place (Hasiotis, 2003). Netto et al. (2007) proposed *Gyrolithes* as a “multipurpose” burrow—the behavioral class polychresichnia—because of the proposed use of the helix in settings with fluctuating salinity as well for microbial gardening or farming. However, if the microbial farming or gardening hypothesis is falsified, then *Gyrolithes* is only a dwelling burrow.

Microbial farming has also been hypothesized to be the behavior represented by the architecturally complex marine trace fossils *Zoophycos*, *Chondrites*, and *Paleodictyon*, which have much more complex morphologies, such as U‐tubes expanded into circular to subcircular or lobate, multilevel spreiten patterns (*Zoophycos*), downward and outward dendritic branching patterns (*Chondrites*) and intricate horizontal tubular patterns with vertical tubes, similar in pattern to a chain‐linked fence (*Paleodictyon*) (Bromley, 1996; Ekdale et al., 1984; Seilacher, 2007; Zhang et al., 2015). If microbes are growing on the walls of these burrows, then it may also be incidental occurrence due to bioirrigation for the purpose of respiration. Direct observations in extant marine helical‐burrowing species of polychaetes and enteropneusts in situ or in a laboratory setting are needed to provide indirect evidence in support of microbial farming‐gardening hypothesis through study of the interaction of the animals feeding on the burrow walls, as well as study of the gut contents of the helical burrow producing animals.

#### 4.1.6 Offspring escape hypothesis

The sole hypothesis involving postnatal parental care proposes that the helix in terrestrial environments, by loosening the soil above the nest, facilitates hatchling escape of neonate monitor lizards (Figure 2); this hypothesis is new and may not apply to any other taxon. There is little doubt that the soil is less resistant in the excavated helix compared to the surrounding soil, which is often compact and firm. In fact, some helices are filled with very loose soils and in some the core of the helix even collapses, leaving a cylindrical section filled with loose soil (JSD, pers. obs.). Hatchling emergence or escape burrows were found for both deep‐nesting species (Doody, McHenry, Brown, et al., 2018a; Doody, McHenry, Durkin, et al., 2018b). Hatchlings excavated escape burrows nearly straight upwards from the nest 2–4 m to the surface, rather than following their mother's soil‐filled burrow. Such deep nesting would challenge hatchlings to emerge through considerable distances of resistant soils. Although emergence burrows were not carefully mapped, at least some of these burrows partly exited through the helix (JSD, unpubl. data, 2023). In short, the helix increases the probability of escaping by reducing the effort required by hatchlings.

Perhaps, in support of this hypothesis, the low clutch size (e.g., 3–8 eggs; Doody et al., 2020) of an animal nesting very deeply might indeed select for parental effort for careful nest excavation to facilitate hatchling escape. While most ground‐nesting reptiles deposit eggs <30 cm below the surface, these lizards nest 2–4 m deep in firm soils, requiring considerable energy for a small group of hatchlings to excavate one emergence burrow. In only one of many nests did we observe two emergence burrows rather than one emanating from the same nest. The position of the helix directly above the nest supports a useful function, but one could ask why the entire burrow is not a helix, although, perhaps, loosening the soil for half the emergence distance is enough to facilitate successful excavation and escape. No other terrestrial species can shed light on this hypothesis because none are known to lay eggs or possess emergence burrows excavated by hatchlings through a helical adult‐excavated burrow.

The recent finding of fossilized neonate *Diictodon* skeletons with adults in burrows assigned to *Daimonelix* suggests that they could have served as brood chambers; whether *Diictodon* bore live young or laid eggs is still under debate (Smith et al., 2021). Pocket Gopher (*Geomys*) nests have also been found associated with helical burrows (Brown & Hickman, 1973; Wilkins & Roberts, 2007). Although the helix could be associated with brooding or eggs in some species other than monitor lizards, the open *Daimonelix* burrows of *Palaeocastor* and *Diictodon* do not support the idea of the helix loosening the soil for neonates as posited by the hatchling escape hypothesis.

Clearly, hatchling escape is likely not a general explanation for helical burrows. This hypothesis could be directly tested by carefully excavating hatchling escape burrows to determine if they typically emanate through the helical portion of the mother's burrow. If they do, measuring the energetic costs of the hatchling escape through resistant (no helix) soil versus less resistant soil (helix) in the laboratory would be ideal. Measuring the energetic cost of the mother's excavation of a helical nesting burrow would also provide context for understanding any energetic benefit to hatchlings.

Perhaps, by analogy, the study of synchronous hatchling emergence from sea turtle nests in foreshore settings in the marine realm can shed some light on the emergence behavior of monitor lizard hatchlings. Studies by Carr and Hirth (1961), Clabough et al. (2022), Miller et al. (2003), and Rusli et al. (2016) among others have shown that new hatchlings work together to dig their way out of the flask‐shaped egg chamber, which is at the base of the body chamber and is excavated and backfilled by the mother. The total depth of the egg chamber controlled by the length of the reach of the turtle's rear paddles excavated at the base of the body pit, which appears to be <1 m deep from descriptions and from photographs (e.g., Bishop et al., 2011; Carr & Hirth, 1961; Miller et al., 2003). Controls on hatchling emergence include the depth of the original chamber, the compaction of the backfilled sediment, as well as the temperature of the sand. Rusli et al. (2016) showed that the energetic cost of escaping through 40 cm of sand varied between 4.4 and 28.3 kJ per individual in the synchronous nest escape, which decreased as the number of individuals in the cohort increased. The reduced energetic cost associated with large cohorts resulted from both a lower metabolic rate per individual and a shortened nest escape time. Rusli et al. (2016) concluded that many hatchlings digging synchronously during nest escape evolved to facilitate rapid nest emergence, which also reduced the energetic cost to each individual.

### 4.2 Construction hypotheses

#### 4.2.1 Falling soil hypothesis

Burrow structures can reflect the energetic cost of burrowing (Vleck, 1981; White, 2001). A new hypothesis, but partly based on previous observations on helical burrowing in scorpions and evidence from vertical burrows in pocket gophers, is that the helices may allow the animals to excavate a steep descent without much soil falling back into the burrow during excavation; that is, the helices would serve to hold much of the newly excavated soil that would otherwise fall back into the burrow terminus as it is removed, impeding or preventing further excavation.

The first line of evidence supporting this hypothesis is found in the relationship in scorpions between climate, soil type, and soil moisture on one hand and on the other burrow depth and number of spirals. Scorpions occupying sandy dry soils in dry climates construct deeper burrows with more spirals than those in wet climates. Polis (1990) hypothesized that by attenuating the burrow angles the spirals would facilitate the vertical movements of scorpions in burrows that are required to be deep enough to reach optimal temperature and humidity. Adams et al. (2016) countered that several species of scorpions construct simple vertical burrows without spirals that descend at 70–90° from the horizontal to 15–10 cm deep. This comparison is confounded by soil type and moisture, however. Compared to those in more mesic environments, scorpion burrows in more arid climates are deeper and possess more spirals (references in Adams et al., 2016). For example, burrows of the scorpion *Urodacus yaschenkoi* in loose sandy soil with little clay content are significantly shallower in depth and angle, and with more spirals, compared to those in damp, low‐lying areas subject to flooding (Koch, 1977, 1978; Shorthouse & Marples, 1980). Similarly, the deepest scorpion burrows with the most spirals in South Africa occur in soils with high sand content and low content of silt, clay, and organic matter, as well as low soil moisture, compared to scorpion burrows in other areas (Abdel‐Nabi et al., 2004).

A second piece of evidence comes from burrows of the pocket gopher *Thonomys bottae*, which excavates lateral tunnels running parallel to the surface but must push excavated soil to the surface to clear the burrows. According to Vleck (1981), “*Thonomys* in cohesive soils often dig nearly vertical laterals and have little difficulty pushing lumps of excavated soil out or plugging the lateral afterward (unpublished data). However, in cohesionless sands like those in the study area, pocket gophers' efficiency in pushing soil declines as slope increases. At steep angles of ascent, much of a load of sand may fall back down the tunnel, increasing the number of trips necessary to push a given amount out. Laboratory observations indicate that *T. bottae* may also have difficulty in plugging the surface openings of vertical tunnels in cohesionless soils. The slope of laterals is probably dictated by soil characteristics and the differential efficiency of pushing soil with changes in slope. Laterals that ascend at shallow angles may be the most efficient solution in sandy soil.”

White (2001) demonstrated that the energetically cheapest method of reaching an appropriate depth is to burrow the shortest possible distance, which would be straight down (i.e., a vertical shaft). However, they also noted that burrow structure may not be determined solely by energetic concerns, and constructing a burrow from the surface at 90° may not be possible. The burrow entrance constructed by the scorpion *U. yaschenkoi* is angled at about 25–30° (Koch, 1978; Shorthouse & Marples, 1980), only marginally shallower than the angle of repose of dry dune sand (32°: Robinson & Seely, 1980). Thus, if burrows were constructed at angles >32°, sand would fall into, and fill the burrow (White, 2001). Beyond the entrance run, the burrows begin to spiral and descend steeply, as the soil becomes moister and more cohesive with increasing depth. Burrows constructed by *U. yaschenkoi* thus minimize both the energy used during burrow construction by descending as steeply as possible, and the energy required for burrow maintenance by constructing an entrance run that is shallower than the angle of repose of dune sand (White, 2001).

As with scorpions, helical burrows of deeply nesting monitor lizards also exhibit a straight, gently sloping entrance run followed by a steeply descending helix (Figure 3; Doody et al., 2015; Doody, McHenry, Brown, et al., 2018a; Doody, McHenry, Durkin, et al., 2018b). The major difference is that the lizard burrows are soil‐filled; the soil is not removed during construction. Thus, White's (2001) calculations of energy expended moving soil out of the burrow would not apply to the lizard burrows because the lizards do not remove the soil (except for the first ~0.5 m straight run). This focuses attention on the digging action by asking: why not excavate straight down or straight at a steep angle of incline? The answer may lie in the ability of the spiral, combined with the lizard's body, in preventing loose, excavated soil from falling back into the burrow terminus. Resisting the effects of gravity by repeatedly removing falling soil would not only incur extra costs, but could prohibit burrow construction.

Meyer (1999) used volumetric calculations to conclude that a helix would cost 36%–61% more effort than a straight burrow. We do not challenge the calculations or logic used by Meyer (1999); rather, we note that those calculations did not consider the cost of repeatedly moving the same soil that falls into the (shifting) burrow terminus during construction. Thus, the need for constructing deep burrows—which apparently evolved to provide moist conditions during the long dry season incubation period in *Varanus* lizards (Doody et al., 2015; Doody, McHenry, Brown, et al., 2018a; Doody, McHenry, Durkin, et al., 2018b)—may have “prompted” helical construction to reduce the energetic cost of repeatedly removing falling soil out of the way, or the falling soil could have prohibited construction altogether. Another factor to consider is the degree of firmness or cohesiveness of the soil in which *Daimonelix* was excavated. If the burrow walls contain scratch marks (sensu Zonneveld et al., 2022), then the sediment was cohesive and much less likely to collapse. This would limit the amount of effort in moving excavated material as long as the matrix did not collapse in on it. Vertical burrows in stiff or firm, cohesive sediment would also stay open; however, the biomechanics of the organism would determine if a vertical burrow was a constructable and/or livable situation (cf., Hasiotis & Mitchell, 1993).

Could the falling soil hypothesis explain helical burrows in other animals? The challenge of constructing a burrow vertically while resisting the effects of gravity on both the body and loosened soil could be general. Even in aquatic burrows the excavated soil must be removed upwards against gravity, and a straight vertical shaft could inhibit or prohibit this. Consider the effort an organism expends in maintaining its position in a vertical burrow excavated in soil, 1–5‐m deep, with appendages sprawled while excavating or maintaining the burrow and removing the material to the surface. An arthropod (e.g., arachnid, crustacean, or hexapod) with 6, 8, or 10 pairs of appendages could accomplish this feat (e.g., Hasiotis et al., 1993; Hasiotis & Mitchell, 1993). Such burrow construction by a tetrapod would be awkward if not impossible for carrying material while removing it from the bottom or maintaining its position in the burrow to maintain the burrow walls (Hasiotis et al., 1999).

A long, gently inclined burrow might incur a lower energetic cost to construct depending on the degree of inclination and distance between the burrow entrance and terminus. Soil profiles consist of horizons each having distinct composition and firmness (e.g., Birkeland, 1999; Brady & Weil, 2002; Kraus, 1999). A gently inclined burrow would increase the probability of spending more time within a horizon with similar composition and firmness, whereas a vertical burrow would have a higher probability of passing more quickly through multiple horizons with different composition and firmness. If an inclined burrow passes from a surface horizon that is relatively loose and contains organics (i.e., A horizon) into a subsurface horizon (E, B, or C horizon) that is firmer and more cohesive, then a higher energetic cost would be incurred. The degree of energetic costs depends on the development and thickness of each horizon, which reflects overall soil formation.

The solution to conserve energy expenditure during construction would seem to be a zigzag or switchback pattern which could eventually “tighten” into a helix. However, some marine organisms construct(ed) helical burrows horizontally (e.g., Dworschak & Rodrigues, 1997; Minter et al., 2008); the falling soil hypothesis fails as an explanation for helical burrows in these species. Similarly, some helical sections in the burrows of the shrimp *Callianassa bouvieri* may be excavated upwards (Dworschak & Pervesler, 1988).

Some skinks construct a switchback style of burrow that mimics a helix (Hasiotis & Bourke, 2006; Hembree & Hasiotis, 2006). These switchbacks come from the main part of the inclined burrow, which is flattened, elliptical in cross‐section, and resembles an upside‐down U or reniform shape (Hasiotis et al., 2004; Hembree & Hasiotis, 2006). A possible function of this switchback structure, which is not visible from the surface of the soil, is to escape the burrow if a predator enters. Likewise, if the hidden switchback burrow opening is discovered, a potential predator might not be able to follow the tortuous path into the main part of the burrow. The switchback may have been a precursor to the helix in burrows, with successive switchbacks being more fully incorporated into a smoother transition of a helical burrows (Figure 4d).

Falling sediment and/or sediment collapse is a less likely explanation for helical burrow construction in marine environments in which they occur. The construction of vertically and horizontally oriented helical burrows is typically conducted in softground and firmground media in shallow marine and deeper marine settings by vermiform animals and crustaceans (e.g., Dashtgard & Gingras, 2012; Gingras, Dashtgard, et al., 2008a; Gingras, Pemberton, et al., 2008b). In intertidal settings, some of the vermiform‐constructed helical burrows have a thick wall lining, which is speculated to help the tube to remain open while water moves through. Crustaceans, in some cases will reinforce their burrows with sediment pellets when the medium is sandy and less cohesive (de Gibert et al., 2006, 2012). However, burrow collapse is unlikely due to their cohesive nature and application of a thin mucus and/or sediment lining along the burrow wall.

Testing the falling soil hypothesis in monitor lizards could involve observing nesting females in captivity to determine how well mothers prevent soil from reaching the burrow terminus; alternatively, recreating a helix in the laboratory with a model lizard could shed light on this ability, as would experiments with humans attempting to construct a helical burrow. Likewise, this can be done with skinks and scorpions in the laboratory and in the field with the natural sediments in which they burrow.

#### 4.2.2 Anticrowding hypothesis

Many investigators have mentioned or addressed the possibility that helical burrowing could reduce crowding and subsequent interference that might otherwise occur if there were multiple burrows with straight runs or ramps within a discrete area (Adams et al., 2016; Doody et al., 2015; Gingras, Dashtgard, et al., 2008a; Gingras, Pemberton, et al., 2008b; Koch, 1978; Martin & Bennett, 1977; Meyer, 1999; Shorthouse & Marples, 1980). For example, Martin and Bennett (1977) supposed that helical burrows in *Palaeocastor* could save horizontal space and avoid neighboring burrows while maintaining a shallow incline, great depth and close packing of burrows. For scorpions, Koch (1978) mentions the avoidance of neighboring burrows under crowded conditions as a possible function, and Shorthouse and Marples (1980) hypothesized that helical burrows might decrease the risk of antagonism or cannibalism by reducing the probability of encounters between scorpions from neighboring burrows. When discussing the function of *Gyrolithes*, Gingras, Dashtgard, et al. (2008a) and Gingras, Pemberton, et al. (2008b) considered likely that the similar helical burrows of thalassinidean shrimp were a response to high population densities.

Meyer (1999) used field data from *Palaeocastor*‐constructed *Daimonelix* to calculate that burrow interference with straight ramps or runs would lead to a low probability of a burrow interfering with another (5%–8%). Adams et al. (2016) suggested that this hypothesis predicts that burrows in dense populations should be more helical than those in sparse populations of hormurid scorpions, yet they noted that simple, vertical shafts occur at similar densities to helical burrows (Harington, 1978). Cambrian *Gyrolithes* examined by Laing et al. (2018) were relatively sparse, which may be due to them being part of larger burrow systems (see Laing et al., 2018, text figure 5b,f).

The anticrowding hypothesis is supported by some evidence of high densities of burrows, but is extremely difficult if not impossible to test directly. An indirect test would be to characterize burrow types at different densities, assuming that the inclusion of helices is a phenotypically plastic behavior (cf. Harington, 1978), or that there has been behavioral evolution among populations leading to disparate burrow morphology.

#### 4.2.3 Vertical patch hypothesis

A third new hypothesis reflects local heterogeneity in media stiffness in the vertical vs. horizontal planes. Each soil horizon tends to be more homogeneous in composition compared to overlying and underlying horizons due to the abiotic and biotic processes that form them (Birkeland, 1999; Brady & Weil, 2002; Hasiotis, 2007; Hasiotis & Platt, 2012; Kraus, 1999). Overall, there is greater heterogeneity vertically in a soil profile than laterally, where the tendency is for the surface (A) horizon to be looser with a greater amount of organic matter compared to the underlying B horizon; i.e., the zone of accumulation of clays and other minerals that make it firmer and more compact. Thus, deep burrowing in the form of a vertical burrow or tightly helical burrow will incur a higher energetic cost to construct the burrow. However, this cost is offset by the benefits of (1) greater relative humidity and soil moisture (microclimate amelioration) combined with (2) greater soil density (firmness and consistency) to ensure a lower chance of soil collapse (i.e., escape of hatchlings). In the exceptional case of monitor lizards, this can also double as better burrow construction in that sediment‐filled burrows result in predator avoidance.

Where shallower optimal or suitable sediment layers predict similar layers directly below, creators constructing helical burrows once those shallower sediment conditions have been discovered would be beneficial, rather than burrow by chance into suboptimal or unsuitable conditions by excavating angular burrows. Relevant conditions could be sediment or soil friability, hardness, grain size, roots, and pre‐existing open burrows, or some biotic factor related to food or farming. In the case of deposit feeding or microbial farming there could be post‐construction benefits (see under each hypothesis). With the monitor lizards, most nesting areas are apparently communal and traditional, possibly due to the reduction in excavating costs associated with constructing burrows in soil already loosed by conspecifics (Doody et al., 2015; Doody, McHenry, Brown, et al., 2018a; Doody, McHenry, Durkin, et al., 2018b).

#### 4.2.4 Biomechanical advantage

A fourth new hypothesis for helical burrows relates to biomechanical advantage. As a burrower excavates, it may be better able in terms of leverage, to remove or rework sediment (soil) with a lateral (side) stroke that results in the burrow bending left or right. This could result in a savings in energy or better efficiency in excavation that could offset the increased effort required, mathematically, to excavate a helical burrow rather than a straight burrow (as calculated by Meyer (1999) for *Palaeocastor* constructing *Daimonelix*; but see the previous Section 4.2.1 on Falling soil hypothesis). This pattern would also result in easier removal of excavated material from the burrow for many tetrapods and arthropods.

In terrestrial settings under vadose zone conditions in the continental realm, helical burrowing in stiffer, more cohesive media (sediment, soil) appears to be a tendency observed by one of us (STH) in burrow construction by spiders and crayfish (also see Hasiotis & Bourke, 2006). For example, in the case of spiders, crayfishes, and tiger beetle larvae excavating in alfisols (illuviated clay‐rich subsurface horizon), a vertical burrow is constructed through the A horizon. However, when the stiffer B horizon is encountered, the direction of the burrow shifts to one side or another or flattens out to form a chamber. In the case of crayfishes, a partial whorl may be produced but then the burrow is continued vertically downward. Such a result might also be expected for tetrapods that construct helical burrows in stiff or firm, cohesive soils in the vadose zone (also see Hembree & Hasiotis, 2006; Riese et al., 2011). The firmness of the soil environment may have led animals to dig in a helical pattern in order to move downward, producing a helical burrow (Figure 4e).

In shallow and in some shelf and deep marine settings, *Gyrolithes* is typically constructed by crustaceans (Uchman & Hanken, 2013) and polychaete worms (Gingras, Dashtgard, et al., 2008a; Gingras, Pemberton, et al., 2008b; Powell, 1977) in subaqueous conditions under which the sediment pore spaces in the benthos are fully saturated. When constructed in firmgrounds, the walls of *Gyrolithes* may contain scratch marks (sensu Zonneveld et al., 2022) as evidence of a firm or stiff medium (Uchman & Hanken, 2013). However, no animal has been observed in the construction of this burrow form, thus, no determination can be made as to if there is a biomechanical advantage in its construction.

*Helicolithus* and *Helicodromites* are also constructed by polychaetes and enteropneusts in subaqueous conditions under which the sediment pore spaces in the benthos are fully saturated (Uchman & Rattazzi, 2023). Some researchers (Dorgan, 2015; Dorgan et al., 2013; Grill & Dorgan, 2015; Law et al., 2014) have proposed that the helically arranged muscle structure and fibers may assist in burrowing by producing peristaltic motion, which also allows for forward and backward movement. These studies also found that left‐ and right‐handed helical muscles wrap around the thorax of worms of all sizes in addition to longitudinal and circular muscles needed for peristaltic movements. Perhaps, the combination of burrowing by dorsoventral muscular forces and the use of the proboscis through fully saturated, heterogeneous sediment may produce helical burrows in vertical, lateral, and horizontal orientation. This potential type of biomechanical advantage is limited only to some groups of vermiform organisms and is not an overarching explanation for helical burrow construction by other invertebrates and vertebrates.

There is currently no strong evidence to support this hypothesis in any taxon other than for *Gyrolithes* and *Daimonelix*‐type burrows occurring in firmgrounds or in soils, respectively. Other burrows, both simple and complex in architecture, also occur in firmgrounds and in soils, however, they do not exhibit helical burrows nor do they have portions of them that are helical. However, Monod et al. (2013) hypothesized that different burrow architecture between taxonomic groups of scorpions was due to behaviors related to morphology: fossorial hormurids are pedipalp burrowers that use the large, often rounded pedipalpal chelae to loosen the soil and carry it out of the burrow, whereas the closely related scorpionoid families are cheliceral burrowers that use their enlarged chelicerae to loosen the soil and then scrape it out of the burrow using the legs and/or metasoma (see references in Monod et al., 2013). Barrass (1963) found the direction of the spiral was related to the asymmetry of the crab's claws such that the males with the major claw on the right exit from burrows that (in the strict sense, as a crustacean burrow) coil counterclockwise, and vice versa. Perhaps, this the production of a helix is more closely related to this morphologic asymmetry in bilaterally symmetrical animals in marine settings.

Testing the biomechanical hypothesis would minimally require observing the digging strokes of decapods and tetrapods and understanding the biomechanics of burrow excavation and preferably involve a comparison of energy required and the efficiency of strokes that would create helical vs. straight burrows. Toots (1963) provided an insightful treatment of the fundamental biomechanical requirements of helical burrow construction by considering the need for asymmetrical digging along the horizontal axis and geotaxis and transverse gravity orientation. This represents a good starting point for exploring the biomechanical underpinnings of constructing a helical burrow, which may provide insights into energetics and construction costs and benefits. For vermiform animals, further observations using a variety of polychaetes and enteropneusts should be made under videography or CT‐tomography while burrowing in sediment of different grain size and media consistency, and evaluated for the burrow morphologies produced.

#### 4.2.5 Additional hypotheses

A few other previous hypotheses mentioned in the literature warrant less attention based on their lack of generality. Koch (1978) proposed that extensive coiling in scorpion burrows could reduce the effects of wind‐blown debris entering the burrow. This is plausible, but scorpions and other terrestrial animals often clear their burrows of debris including blown sand, caved‐in sand, and vegetation (Shorthouse & Marples, 1980). Helical burrows produced by marine organisms may help keep the burrow free of sediment debris. Another hypothesis, raised for *Gyrolithes*, suggests that the helical burrow promotes anchoring of the tracemaker in the burrow in high‐energy environments (Gingras, Dashtgard, et al., 2008a; Gingras, Pemberton, et al., 2008b; also Laing et al., 2018). However, the *Gyrolithes* studied by Moosavizadeh and Knaust (2021) reflects a low‐energy environment. Finally, some male ghost crabs (*Ocypode*) may construct spiral burrows for courtship (Clayton, 2005; Schober & Christy, 1993). However, only one species has been shown to construct burrows with more than one whorl, at least some of the burrows of *Ocyopde ceratophthalmus* exhibit two whorls (Parenzan, 1931, in: Eshky, 1985; Fellows, 1966, in: Vannini, 1980). Some authors have hypothesized that helical burrows serve as domiciles that protect the burrow inhabitants (e.g., Laing et al., 2018). We did not consider this hypothesis because it is oversimplified (i.e., all burrows provide protection to the constructors) and cannot in itself explain the evolution, benefits, or function of helical structure above and beyond what the aforementioned hypotheses (e.g., antipredator) attempt to explain.

### 4.3 Potential evolutionary sequence of deep, helical burrowing behavior in some monitor lizards

We can reconstruct the putative evolution of nesting behavior in these species using the discussions in previous sections. Large monitor lizards that lay large eggs that require long incubation periods (6–9 months; Horn & Visser, 1989, 1997) that must stretch over dry seasons in species inhabiting arid areas, at least in Australia (Doody et al., 2015; Doody, McHenry, Brown, et al., 2018a; Doody, McHenry, Durkin, et al., 2018b). These species have evolved the behavior of nesting much deeper than any other reptile (Doody et al., 2014, 2015; Doody, McHenry, Brown, et al., 2018a; Doody, McHenry, Durkin, et al., 2018b). The cheapest way, energetically, *with regard to distance only*, for a monitor lizard to nest 2, 3, or 4 m deep is to construct a vertical tunnel straight below the site they have chosen (White, 2001, referring to scorpions). However, a lizard cannot excavate a burrow straight down because the soil continues to fall in on itself. For the lizard to remove the soil from the burrow once they are deeper than 1 m is effectively impossible because the soil would need to be thrown upwards out of the burrow a considerable distance with efficiency. To our knowledge, monitor lizards cannot carry or transport soil other than kicking or dragging it on the surface with their limbs, head, and neck. So, a deep, straight vertical burrow is physically impossible because the creator could not get the loose soil out of the way to allow continued burrow construction.

A physically manageable but more energetically expensive (distance‐wise) approach would be to excavate an inclined (straight) burrow run at an angle that would prevent soil from falling back down once loosened. The mean incline for *V. panoptes* burrow entrances is 8° (Doody et al., 2015). If the burrow is to be 3 m deep, with an angle of 8°, solving for the opposite side of a right triangle yields a horizontal distance of the nest from the burrow entrance of 19 m (13 m if 2 m deep, 26 m if 4 m deep). This is a considerable distance from where the mother selected a suitable patch of soil, creating risk that she might encounter more resistant soils that would be more costly to burrow through. In support, both *V. panoptes* and *V. gouldii* nest communally and traditionally, apparently taking advantage of soil loosened by conspecifics by nesting in a discreet area of soil that is softer than the surrounding area (Doody et al., 2015). Increasing the angle of incline (steeper) would decrease the horizontal distance of the nest from the burrow entrance, but at some point, the incline allows soil to fall back into the burrow. As noted earlier, continually removing soil that is falling back into the burrow is energetically expensive and probably impossible at depths greater than 1 m. This cost could be large enough to offset or even outweigh the cost of constructing a helix (calculated by Meyer, 1999). Steeper inclines would at some point be prohibitive (as with the vertical burrow above).

A possible solution is the construction of a helix, which is physically manageable, and possibly energetically equivalent or superior to a straight incline and would bring the creator directly down into the intended nesting area with loosened soil. Perhaps, there are intermediates that resemble a zigzag or switchback pattern; these could eventually have “tightened” into a helix. Stopping the falling soil might be especially needed for monitor lizards because they do not remove the soil from the burrow, except for the first 0.5 m of the entrance run.

## 5 CONCLUSIONS

Our review is the first to consider all taxa when addressing the evolution and function as well as the costs and benefits of helical burrowing in animals. Our examination of the fit of 10 hypotheses to numerous living and extinct taxa failed to find compelling evidence for any one general hypothesis for why animals construct helical burrows. Only two hypotheses—antipredator and biomechanical advantage—cannot be rejected for any species, although six of the hypotheses cannot be rejected for most species (possible in 86%–100% of species). Thus, one or more of these could explain the behavior of helical burrowing in most species. Four of these six are construction hypotheses, raising the possibility that helical burrowing might have evolved without providing postconstruction benefits. Our analysis did eliminate four hypotheses of increased drainage, deposit feeding, microbial farming, and offspring escape as explanations for helical burrowing behavior in the majority of taxa (possible in only 5%–48% of species). The extended phenotype of helical burrowing probably evolved for a variety of reasons. Further observations of helical burrowing in different biotic and abiotic contexts, and in particular, experiments, could in some cases eliminate or provide support for some of the hypotheses, while other hypotheses are difficult to test, or not directly testable.

## AUTHOR CONTRIBUTIONS

**J. Sean Doody:** Conceptualization (lead); data curation (equal); investigation (equal); methodology (lead); writing – original draft (lead); writing – review and editing (equal). **Stephen T. Hasiotis:** Data curation (equal); investigation (equal); writing – original draft (supporting); writing – review and editing (equal). **Shivam Shukla:** Data curation (equal); resources (equal); writing – original draft (supporting); writing – review and editing (equal).

## Data Availability

All data are contained within this manuscript and its associated tables.
